# A comparison of the nutrient intake of a community-dwelling first-episode psychosis cohort, aged 19–64 years, with data from the UK population

**DOI:** 10.1017/jns.2015.18

**Published:** 2015-08-20

**Authors:** Kevin Williamson, Karen Kilner, Nicola Clibbens

**Affiliations:** 1 Rotherham Early Intervention in Psychosis Service, Rotherham Doncaster and South Humber NHS Foundation Trust, 144A Aughton Road, Swallownest Court, Swallownest, Sheffield S26 4TH, UK; 2 Sheffield Hallam University, P102 Montgomery House, 32 Collegiate Crescent, Sheffield S10 2BP, UK; 3 Sheffield Hallam University, 36 Collegiate Crescent, Sheffield S10 2BP, UK

**Keywords:** First-episode psychosis, Dietary assessment, Micronutrient deficiencies, Metabolic syndrome, NHS, National Health Service, NME, non-milk extrinsic, RNI, reference nutrient intake

## Abstract

Psychosis increases the risk of CVD, obesity and type 2 diabetes and reduces life expectancy. There are limited data comparing the dietary habits of community-dwelling first-episode psychosis sufferers – with autonomy over diet – and the general population. The data represent the retrospective evaluation of nutritional data collected between 2007 and 2013 from 143 individuals from the UK population receiving treatment for first-episode psychosis. Differences in mean nutrient intakes between the study cohort and the national sample were tested for statistical significance using independent *t* tests, incorporating Satterthwaite's correction where required. Mean total energy intake was lower for males (*P* = 0·049) and higher for females (*P* = 0·016) in the cohort than in the corresponding subgroups of the national sample. Females in the study cohort consumed 12·9 (95 % CI 4·3, 21·5) g more total fat per d, whilst males consumed 7·7 (95 % CI 0·5, 14·9) g less protein per d than the national sample. Males in the study also showed significantly lower mean intakes than nationally of folate, Fe, Se, vitamin D and Zn, but not vitamin C. The proportion of individuals not meeting the lower reference nutrient intakes, particularly for Se (males 54·0 % and females 57·1 %) and for Fe amongst females (29·6 %), is cause for concern regarding potentially severe deficiencies. Further exploration of dietary habits within first-episode psychosis is warranted to assess whether individuals make beneficial dietary changes for their physical and mental health and wellbeing following dietary change intervention. It would also be pertinent to assess any correlation between diet and mental health symptomology.

Severe mental illness, such as schizophrenia, increases the risk of CVD, obesity and type 2 diabetes and reduces life expectancy^(^^1^^–^^7^^)^. Schizophrenia is a condition characterised by episodes of psychosis, hallmarked by an alteration of perception, thoughts, mood or behaviour^(^^8^^)^. There are several key factors potentially affecting the food consumption patterns in those affected, including socio-economic status^(^^9^^,^^10^^)^, an illness-induced lack of motivation and a sedentary lifestyle compared with the general population^(^^5^^,^^11^^–^^13^^)^.

A recent systematic review reporting thirty-one published studies on dietary patterns in schizophrenia cited only four which related to first-episode psychosis^(^^14^^)^. Whilst these^(^^15^^–^^18^^)^ – all case–control studies – provided useful information, it is unclear whether the study groups reported had control over their own dietary choices. Autonomy over dietary choice is important because acutely psychotic individuals in the community may fail to eat meals as a consequence of the chaos of psychotic symptoms^(^^19^^)^, and could help determine which aspects of the diet would benefit from dietary change intervention. There are limited data comparing the dietary habits of community-dwelling first-episode psychosis sufferers with the general population or with an optimum nutrient intake. One study^(^^18^^)^, for example, assessed dietary intake through recording food frequency over the previous 1 year as part of the Health and Lifestyle 2 (HAL2) questionnaire. This assessed habitual frequency, may inaccurately reflect current intake, in light of recent mental health problems, particularly if the individual has a short duration of psychosis.

Antipsychotic medication, which whilst improving symptomology, is known to cause carbohydrate craving^(^^20^^,^^21^^)^ and hyperphagia in individuals^(^^22^^–^^27^^)^. Animal models demonstrate that abdominal fat deposition can occur whilst undergoing treatment with antipsychotic medication, irrespective of a corresponding change in energy intake^(^^28^^)^. This effect, found to be reversible following the cessation of the pharmacological agent^(^^28^^)^, emphasises the need for robust nutritional assessment and intervention in this population.

Not only are macronutrients of significance in severe mental illness, micronutrients are also essential cofactors in mental function, via a potential range of mechanisms, including: deficient methylation, impaired mitochondrial function, alteration of gene expression and the impaired growth and development of neurons^(^^29^^)^. Folate is an integral component of methylation processes, notably the formation of neurotransmitters controlling mood^(^^30^^,^^31^^)^. Logically, therefore an adequate folate intake must be advocated in individuals with severe mental illness. Likewise, there is emerging evidence for vitamin D as a functioning neurosteroid^(^^32^^)^, implicating it in the development and general function of brain homeostasis. Evidence indicates that vitamin D helps reduce Ca^2+^ levels in the brain, inhibits glutathione metabolism and protects against reactive oxygen and nitrogen species^(^^33^^)^. Additionally, vitamin D has been specifically associated with psychotic disorders. Individuals with schizophrenia were found to have significantly lower plasma levels of vitamin D^(^^34^^,^^35^^)^. Whilst some consider that this could be linked to dietary habits^(^^34^^)^, others, however, found that the diets were not lacking in Ca-rich dairy foods^(^^35^^)^. In addition to these studies conducted in adult cohorts, there is evidence of vitamin D deficiency associated with the prevalence of psychotic features in adolescents^(^^36^^,^^37^^)^. A large proportion of vitamin D is sourced from sunlight and this could well be lacking in those with psychosis through limited outdoor exposure brought about from the social isolation and sedentary lifestyle often associated with psychosis^(^^38^^)^. It is important thus to ensure that a baseline dietary intake is established and then education around optimal dietary intake be provided, where warranted.

Antioxidant systems are especially important for those with psychosis. There is evidence that oxidative damage to neurons has been associated with, and may therefore contribute to, the pathophysiology of schizophrenia^(^^39^^–^^41^^)^. Micronutrients are involved in the production of several of the body's antioxidant systems. The importance of Fe, Cu and Mn has been cited as transition metals that can cause free radical damage^(^^42^^)^. Se is an essential component of the glutathione peroxidase group^(^^43^^)^. An alteration from physiologically optimal Mn, Cu, Zn, Se and Fe levels may play a role in the development of schizophrenia and that dietary supplementation or dietary improvement may be effective through the corresponding increase in antioxidant activity^(^^44^^)^.

The aim of the present paper was to begin to address the paucity of nutritional information and present population comparative data for key macro- and micronutrients from community-dwelling individuals with first-episode psychosis from the UK population who are responsible for their own diets.

## Materials and methods

### Study design and population

The primary data described represent the retrospective evaluation of nutritional data collected from individuals receiving treatment for first-episode psychosis. These subjects are part of the UK population, have a diagnosis of first-episode psychosis and are undergoing treatment from the National Health Service (NHS). No individuals displaying acute symptoms of psychosis, as determined by a health professional, were offered a nutritional assessment as this was not a health care priority for them at that time. Individuals in the psychosis study series, to be comparable with the national sample, were all community-dwelling (not hospital residents) and were either directly in control of their food purchases, or household foods were purchased via a partner or relative, at the time of the survey.

The cohort under study (*n* 143) comprises eighty-seven males and fifty-six females, who were asked by a health professional (not the nutritionist) from the Early Intervention in Psychosis service if they would like a nutritional assessment. All cases thus consented to, and completed, the nutritional assessment voluntarily as part of their treatment within the Early Intervention Service. The subjects ranged in age from 19 to 64 years. The comparative data described herein are published data collected from a sample of the UK general population by the Department of Health's Food Standards Agency (The National Diet and Nutrition Survey 2008–2011). Subject details of this comparative study series have been described elsewhere^(^^45^^)^, though in summary: full datasets were compiled on nutrient intake from 519 male and 667 female participants aged 19–64 years, that consensually opted to take part in the National Study.

### Ethics

This study was conducted according to the guidelines laid down in the Declaration of Helsinki and all procedures involving patients were approved by the NHS host institution's Research Governance Department. (This study did not require ethical approval.) Verbal informed consent was obtained from all patients relating to their participation in a nutritional assessment for the purposes of their healthcare needs. Verbal consent was witnessed and formally recorded in their patient notes. The dataset was fully anonymised before statistical analysis was conducted. Individuals in the psychosis study series were informed throughout the process that they could stop participation in the nutritional assessment at any time, without the need to give any reason and without any detrimental effect on their current or future health care. For the national sample, written consent was obtained from all adult participants.

### Dietary data collection and analysis

The same method was used to collect dietary data from the psychosis study series and the national sample, with all foods and beverages consumed in a food diary logged over four consecutive days. In the psychosis study series, a face-to-face interview was given to introduce the diary to the participants who completed this over the subsequent 4 d. Data from the first-episode psychosis study series were collected between 2007 and 2013, as part of routine health care within the psychosis service, from a population of individuals maintained on the caseloads of two Early Intervention in Psychosis services, following their first psychotic episode. The food diary, once completed by the participants, was returned to a health professional in the Early Intervention in Psychosis Service.

Analysis of the food diaries for comparison of nutrient profile was via NetWISP dietary analysis software (Tinuviel Software), for the psychosis study series. The key nutrients presented in this publication include total energy, non-milk extrinsic (NME) sugar, total fat, saturated fat, protein, carbohydrate, vitamin C, vitamin D, folate, Fe, Se and Zn.

### Statistical analyses

Statistical analyses of the comparison between both datasets were carried out using SPSS for Windows v20 (SPSS Inc.). For selected nutrients, descriptive statistics (mean, median, standard deviation, 97·5th and 2·5th percentiles) were calculated, in line with those published for data from the National Diet and Nutrition Survey. Differences in mean nutrient intakes between the study series and the national sample were tested for statistical significance using independent *t* tests, incorporating Satterthwaite's correction where required. Micronutrient intakes in the study series were also compared with established reference nutrient intakes (RNI) and lower RNI developed by the Scientific Advisory Committee on Nutrition from the 1991 Committee on Medical Aspects of Food and Nutrition Policy report citing dietary reference values^(^^46^^)^.

## Results

### Total energy and macronutrient intake

Wide variability is evident in both groups for all intake variables. In the study series, average total energy intake is lower for males (*P* = 0·049) and higher for females (*P* = 0·016) than in the corresponding subgroups of the national sample (Table 1). Males’ mean total energy intake is 646 (95 % CI 2·1, 1215) kJ less in the study series than nationally, while for females the difference is 650·6 (95 % CI 119·7, 1181·6) kJ more in the study series.

**Table 1.** Summary statistics for daily energy and macronutrient intakes

		Energy (kJ/d)		Total fat (g/d)		Saturated fat (g/d)		Protein (g/d)		Carbohydrate (g/d)		NMES (g/d)	
		Sample	National	Sample	National	Sample	National	Sample	National	Sample	National	Sample	National
All	Mean	7962·2	7690·2	77·2	68·3	27·0	25·1	74·8	74·2	230·6	219·0	66·3	57·2
	P*	0·224		<0·001		0·050		0·801		0·155		0·088	
	Median	7439·2	7405·7	73·7	65·8	25·0	23·8	74·1	71·8	211·5	211·0	49·9	47·8
	sd	2690·3	2523·0	30·1	27·1	12·2	11·3	27·6	27·3	92·5	72·0	62·0	38·4
	97·5th Percentile	14267·4	13112·7	151·3	127·9	53·4	50·9	141·4	125·1	443·7	365·0	204·7	150·7
	2·5th Percentile	3765·6	3468·5	32·1	22·8	7·4	7·5	30·9	34·0	99·1	91·0	5·8	7·2
Males	Mean	8359·6	8970·5	80·0	78·9	28·2	28·8	78·3	86·0	240·9	250·0	75·5	68·9
	P*	0·049		0·736		0·651		0·036		0·413		0·408	
	Median	7911·9	8807·3	77·4	77·2	26·5	27·6	79·9	84·4	216·4	246·0	53·5	63·5
	sd	2815·8	2640·1	29·0	28·0	11·4	11·7	30·2	32·0	104·8	76·0	72·2	41·5
	97·5th Percentile	13217·3	14719·3	146·6	143·1	53·6	54·0	144·7	142·6	495·6	407·0	243·6	168·5
	2·5th Percentile	3765·6	4368·0	32·2	30·4	8·5	9·4	29·1	43·8	97·3	120·0	6·4	9·2
Females	Mean	7342·9	6694·4	72·8	59·9	25·2	22·1	69·4	65·0	214·7	195·0	52·1	48·1
	P*	0·016		0·004		0·098		0·153		0·017		0·396	
	Median	6836·7	6568·9	69·5	57·6	21·9	20·4	71·3	64·2	200·2	193·0	39·7	41·4
	sd	2384·9	1903·7	31·5	23·1	13·3	9·9	22·2	18·4	67·0	59·0	38·0	33·1
	97·5th Percentile	14267·4	10773·8	173·0	108·7	71·3	44·0	122·0	104·0	367·0	321·0	139·7	126·8
	2·5th Percentile	4192·4	3100·3	16·7	20·2	4·5	6·4	17·0	31·9	69·9	86·0	2·3	5·6

NMES, non-milk extrinsic sugar.

* Independent *t* test comparing means of study cohort and national sample.

Amongst the macronutrients, the most notable difference is in mean total fat intake amongst females (*P* = 0·004); females in the study series consume, on average, 12·9 (95 % CI 4·3, 21·5) g more total fat per d than those in the national sample. Indeed, at all points in the distribution, total fat consumption appears considerably higher for females in the study series than in the national sample. Amongst males, a lower mean protein consumption is evident in the study series (*P* = 0·036); males in the study consume an average 7·7 (95 % CI 0·5, 14·9) g less protein per d than those in the national sample.

### Micronutrients

In contrast to macronutrient intake, several micronutrients have significantly lower reported intakes in the study group, some markedly so, particularly amongst males.

Vitamin D intake was, on average, 2·3 µg/d in males, *v.* 3·1 µg/d in the general population and females consumed 1·6 *v.* 2·6 µg/d in the general population (Table 2). These figures represent an intake of only 74 and 62 % of the population intake, respectively, and there is mounting evidence that the population does not consume sufficient vitamin D^(^^47^^)^.

**Table 2.** Summary statistics for daily micronutrient intakes

		Vitamin C (mg/d)		Vitamin D (μg/d)		Folate (μg/d)		Fe (mg/d)		Se (μg/d)		Zn (mg/d)	
		Sample	National	Sample	National	Sample	National	Sample	National	Sample	National	Sample	National
All	Mean	77·2	85·7	2·1	2·8	220·9	260·0	10·3	10·7	41·8	47·0	8·3	8·7
	P*	0·168		<0·001		<0·001		0·270		0·004		0·186	
	Median	56·0	69·8	1·5	2·3	199·0	244·0	9·7	10·4	39·0	44·0	7·7	8·4
	sd	85·7	67·2	2·2	2·1	113·0	109·0	4·4	3·8	23·9	21·0	3·2	3·1
	97·5th Percentile	404·2	239·9	9·5	8·3	552·0	525·0	22·3	19·2	96·4	92·0	16·5	15·6
	2·5th Percentile	4·6	13·4	0·1	0·5	74·0	96·0	3·8	4·4	9·6	19·0	2·7	3·8
Males	Mean	77·9	88·4	2·3	3·1	230·0	298·0	10·6	12·0	44·4	53·0	8·6	9·9
	P*	0·237		0·006		<0·001		0·003		0·001		0·001	
	Median	53·0	72·7	1·7	2·6	193·0	280·0	9·4	11·7	40·0	50·0	8·3	9·7
	sd	94·4	73·6	2·6	2·3	125·0	127·0	4·8	4·2	26·3	23·0	3·3	3·4
	97·5th Percentile	512·4	261·8	14·0	9·2	629·0	618·0	23·8	21·4	119·8	101·0	17·5	17·9
	2·5th Percentile	5·4	15·0	0·2	0·5	80·0	111·0	4·1	5·2	10·8	23·0	2·9	4·4
Females	Mean	76·2	83·6	1·6	2·6	206·0	231·0	10·0	9·7	37·7	43·0	7·8	7·7
	P*	0·395		<0·001		0·037		0·555		0·049		0·734	
	Median	61·0	68·4	1·3	2·1	212·0	220·0	10·0	9·5	35·5	39·0	7·2	7·5
	sd	71·0	61·7	1·3	1·8	91·0	82·0	3·8	3·0	19·1	18·0	3·1	2·5
	97·5th Percentile	375·4	227·3	5·3	7·5	470·0	426·0	20·1	15·9	96·6	87·0	15·6	13·2
	2·5th Percentile	1·7	12·9	0·0	0·4	41·0	92·0	2·3	4·1	4·4	18·0	1·6	3·5

* Independent *t* test comparing means of study cohort and national sample.

Males in the study show significantly lower mean intakes than nationally of all the micronutrients considered, except vitamin C. This may be consistent with their lower intakes of protein and total energy noted previously.

Table 3 shows the numbers and percentages of individuals in the study whose micronutrient intakes meet recognised RNI/lower RNI and Table 4 presents the proportion of participants with average daily intakes of vitamins and minerals below the lower RNI. For the majority of micronutrients the study series exhibit a higher proportion of deficiency than the general population.

**Table 3.** Comparison of study subjects’ micronutrient intakes with reference nutrient intake (RNI) and lower RNI (LRNI)*

	Vitamin C				Folate				Fe				Se				Cu				Zn			
	Males		Females		Males		Females		Males		Females^†^		Males		Females		Males		Females		Males		Females	
	*n*	%	*n*	%	*n*	%	*n*	%	*n*	%	*n*	%	*n*	%	*n*	%	*n*	%	*n*	%	*n*	%	*n*	%
At or above RNI	56	64·4	39	69·6	41	47·1	30	53·6	55	63·2	3	5·6	10	11·5	6	10·7	22	25·3	13	23·2	32	36·8	31	55·4
At or above LRNI but below RNI	27	31	13	23·3	36	41·4	19	33·9	28	32·2	35	64·8	30	34·5	18	32·2	65	74·7	43	76·8	41	47·1	19	33·9
Below LRNI	4	4·6	4	7·1	10	11·5	7	12·5	4	4·6	16	29·6	47	54	32	57·1	‡	‡	‡	‡	14	16·1	6	10·7
Total	87	100	56	100	87	100	56	100	87	100	54	100	87	100	56	100	87	100	56	100	87	100	56	100

* There are currently no recommended intakes for vitamin D.

† Aged 50 years or under only. Two females aged over 50 years had levels above the RNI for their age group.

‡ There is currently no LRNI for Cu.

**Table 4.** Proportion of participants with average daily intakes of vitamins and minerals below the lower reference nutrient intake

	Males		Females	
	Study series (*n* 87) (%)	National sample (*n* 519) (%)	Study series (*n* 56) (%)	National sample (*n* 667) (%)
Vitamin C	4·6	0·6	7·1	1·2
Folate	11·5	2·1	12·5	3·3
Fe	4·6	1·5	28·6	28·8
Se	49·4	25·4	57·1	52·0
Zn	16·1	8·3	10·7	4·5

## Discussion

The data represent the nutritional habits of those with a first-episode psychosis. Whilst the duration of untreated psychosis may be variable, the dietary intake of these individuals well represents a first episode of psychosis study series at the baseline of admission to services due to the validated methods used. It is interesting to note that the total daily energy intake in males is within recommended guidelines^(^^46^^)^, yet is lower than the general population^(^^45^^)^. This could be due to missed meals, which has previously been reported in those with acute psychosis^(^^19^^)^ and is within recommended guidelines^(^^46^^)^. Female total energy intake contrasts with the males, as although it too is within current guidelines^(^^46^^)^, it is higher than the national average, as too is their fat intake. The reasons for this are unclear and warrant further research. It is known that stress, such as that brought on by illness, could culminate in stress-induced emotional eating^(^^48^^,^^49^^)^, or it could be a craving for carbohydrates as a response to commencement of antipsychotic medication, albeit within only a few weeks of initiation^(^^50^^)^. Stress has been hypothetically linked to the metabolic abnormalities seen in first-episode psychosis due to evidence of greater stress reported throughout the early and pre-illness phases for individuals^(^^14^^)^.

This finding of increased total energy and carbohydrate intake, particularly in females, within first-episode psychosis concurs with a previous study of those with schizophrenia^(^^51^^)^. When nutrient intakes of community-dwelling schizophrenic individuals in the USA were compared with those of the National Health and Nutrition Examination Study (NHANES) dataset^(^^51^^)^, total carbohydrate intake was significantly higher than that of the general population. The total energy intake reported was again significantly higher than that of the general population, with the largest difference reported in females^(^^51^^)^. A higher fat intake and lower fibre intake relative to the general population have also been reported from a cohort of 102 middle-aged, community-dwelling individuals with schizophrenia^(^^1^^)^. One study reported a lower fat intake in the schizophrenia group, compared with the controls^(^^52^^)^.

Of thirty-one published studies from schizophrenic cohorts, 44 % cited an increased intake of fat; however, the differences observed in those studies could be due to the enduring psychosis and potential hyperphagic effect of antipsychotic medication^(^^20^^,^^21^^)^. Published case–control studies of first-episode psychosis service users have found only one macronutrient – a higher intake of saturated fat – to be higher than that of the general population^(^^15^^)^, which was found to worsen post-antipsychotic treatment for 6 months^(^^16^^)^, whilst others found no difference^(^^17^^,^^18^^)^. The proportions of individuals not meeting the lower RNI, particularly for Se (males 54·0 % and females 57·1 %) and for Fe amongst females (29·6 %), is cause for concern regarding potentially severe deficiencies. In this study the suboptimal micronutrient deficiencies, particularly in males, are concerning because this may indicate that whilst the diet is broadly supplying the energy needs of the individual, the quality of food supplying that energy is substandard. This could have other implications for health, particularly in light of the evidence that micronutrients may be specifically linked to mental health.

Carbohydrate intake is higher in females, although there is no difference between NME sugar intakes, whereas another study has found that those with schizophrenia (not limited to first-episode psychosis) have a diet that is significantly higher in sugar^(^^53^^)^. If this trend toward increased sugar consumption being correlated to increased duration of psychosis was to be repeated in further studies it could have implications, first, for healthcare provision, and moreover the recommendations for sugar consumption in this population subgroup. It is also noteworthy that the study cohort's NME sugar intake was not significantly higher in both sexes than the population, but higher than the current recommendation for both males (75·5 *v.* 55 g/d) and females (52·1 *v.* 45 g/d)^(^^46^^)^. NME sugar consumption has decreased in the general population in the UK in the last decade; the intake of the population, based upon the 2008/2009 rolling assessment programme data, indicates that it is still higher than recommended by the Food Standards Agency^(^^45^^)^. Despite this, intakes are higher than recommended and this reinforces the suggestion that dietary education is warranted, to help reduce sugar intake – or at least prevent an increase – in light of links between sugar and markers of the metabolic syndrome, and the higher prevalence of physical co-morbidities in those with schizophrenia^(^^1^^–^^7^^)^. A high NME sugar intake was found to be associated with type 2 diabetes in one meta-analysis^(^^54^^)^ and again more recently^(^^55^^)^. When the effect of sugar consumption is measured in terms of the glycaemic load, it has reportedly led to an increased CHD risk^(^^56^^)^, which was not mirrored by the total carbohydrate intake. In a review published over a decade ago, high-sucrose diets, when consumed excessively, increased the incidence of hypertriacylglycerolaemia in obese individuals^(^^57^^)^. Further convincing evidence shows that NME sugar in sugar-sweetened beverages contributes to weight gain and obesity^(^^54^^)^. It is also possible that individuals are aware of key public health advice relating to nutrition and the association with metabolic co-morbidities, such as type 2 diabetes and obesity. Further work in this area should consider the measurement of the nutritional knowledge of community-dwelling individuals with first-episode psychosis.

The findings presented here indicate that even within 12 weeks of diagnosis with first-episode psychosis, diets are nutritionally poorer than the population. There are thus pertinent public health issues due, first, to the literature that links nutrients to health in the general population and then moreover to schizophrenia's association with an increased risk of developing physical co-morbidities^(^^15^^,^^58^^–^^61^^)^. This cannot be dissociated from the continued disparity that exists in the life expectancy of those with severe mental illness^(^^2^^,^^7^^)^.

There are several potential consequences of a low micronutrient intake on first-episode psychosis individuals. First, does a potential link exists between their lack of micronutrients and the precipitation of their mental illness? Second, could this micronutrient-deficient diet be hampering their recovery, specifically through a neurotransmission-mediated response to prescribed medications? These processes require key nutrients, including folate, Se and Fe, to effect these changes. Furthermore, as the physiological requirement for vitamin D is met through diet and the duration and intensity of the exposure to sunlight, a chronic deficiency is plausible amongst those with psychosis, due to a possible co-morbid social phobia^(^^62^^)^ and consequent lack of outdoor exposure.

As part of the nutritional education of those with first-episode psychosis, it is prudent to consider the nutritional quality of daily energy sources. A plan of regular nutritional assessments is advisable to ensure that this situation is monitored, in light of the evidence that those with chronic or relapsing schizophrenia have significantly higher macronutrient intake compared with controls and are more likely to develop the metabolic syndrome than the general population.

### Limitations

This paper compares the mean intake of a self-selected study series with first-episode psychosis with the general population. Whilst the study series may be comparable with the sample in the National Diet and Nutrition Survey in as far as these individuals were also self-selecting, this may not be fully representative of the population's dietary intake.

Questions may also arise over the validity of self-reported dietary intakes in general; however, in some cases food diaries were backed up by members of the individuals’ care networks (partners or family members) and the concordance of total energy intake with the general population shows that drawing comparisons from the two datasets is valid. The generalisability of these results is limited though because no physiological measures, e.g. haematological or fingernail samples, were taken from the psychosis cohort to improve the validity of the participant reporting in the food diaries and any incomplete or inaccurate micronutrient data from the food composition tables. This would be advantageous in any future study of this type.

It is limiting that no information was available relating to the socio-economic status or food purchasing patterns of the psychosis cohort. Socio-economic status has long been linked to food purchasing patterns, nutrient consumption^(^^63^^)^ and thus possibly health outcomes, yet whilst that data would have been useful, it is important to note that psychosis can affect any individual, irrespective of socio-economic or lifestyle factors. Likewise, a measure of other variables that affect dietary intake behaviour, such as cooking ability, influence of family and peers and knowledge of the links between nutrition and health^(^^64^^,^^65^^)^ was not recorded for the psychosis cohort; however, this would warrant further investigation. Prescribed antipsychotic medication has also been linked to an alteration of food intake and metabolism^(^^20^^–^^28^^)^; thus a limitation of this study, and one that would warrant further consideration, is the lack of data pertaining to both the initiation and dosage of treatment with antipsychotic medication and any effect that may have on the dietary habits of those with psychosis.

### Conclusion

Further exploration of dietary habits with first-episode psychosis would be warranted to assess whether individuals make dietary changes that would benefit their physical and mental health and wellbeing following dietary change intervention. It would also be pertinent to assess any relationship between diet and mental health symptomology.
